# Supplementary material for: Distribution and Maturity of Medial Collagen Fibers in Thoracoabdominal Post-Dissection Aortic Aneurysms: A Comparative Study of Marfan and Non-Marfan Patients
Source: Int J Mol Sci. 2024 Dec 24;26(1):14. doi: 10.3390/ijms26010014 (PMC11720456; doi:10.3390/ijms26010014)
Supplement: Supplementary file 1 [file ijms-26-00014-s001.zip › ijms-3368018-supplementary.pdf]

Descending thoracic aorta	Suprarenal aorta	Infrarenal aorta	Age (years)	Marfan
48,6	27,8	61,6	35	No
17,7	18,1	31,1	35	Yes
19,8	25,2	48,9	57	No
24,7	27,8	68,3	57	No
15,9	18,1	28,2	58	Yes

Supplementary Table S1. Percentage of total collagen in the aortic media in 5 age-matched patients.

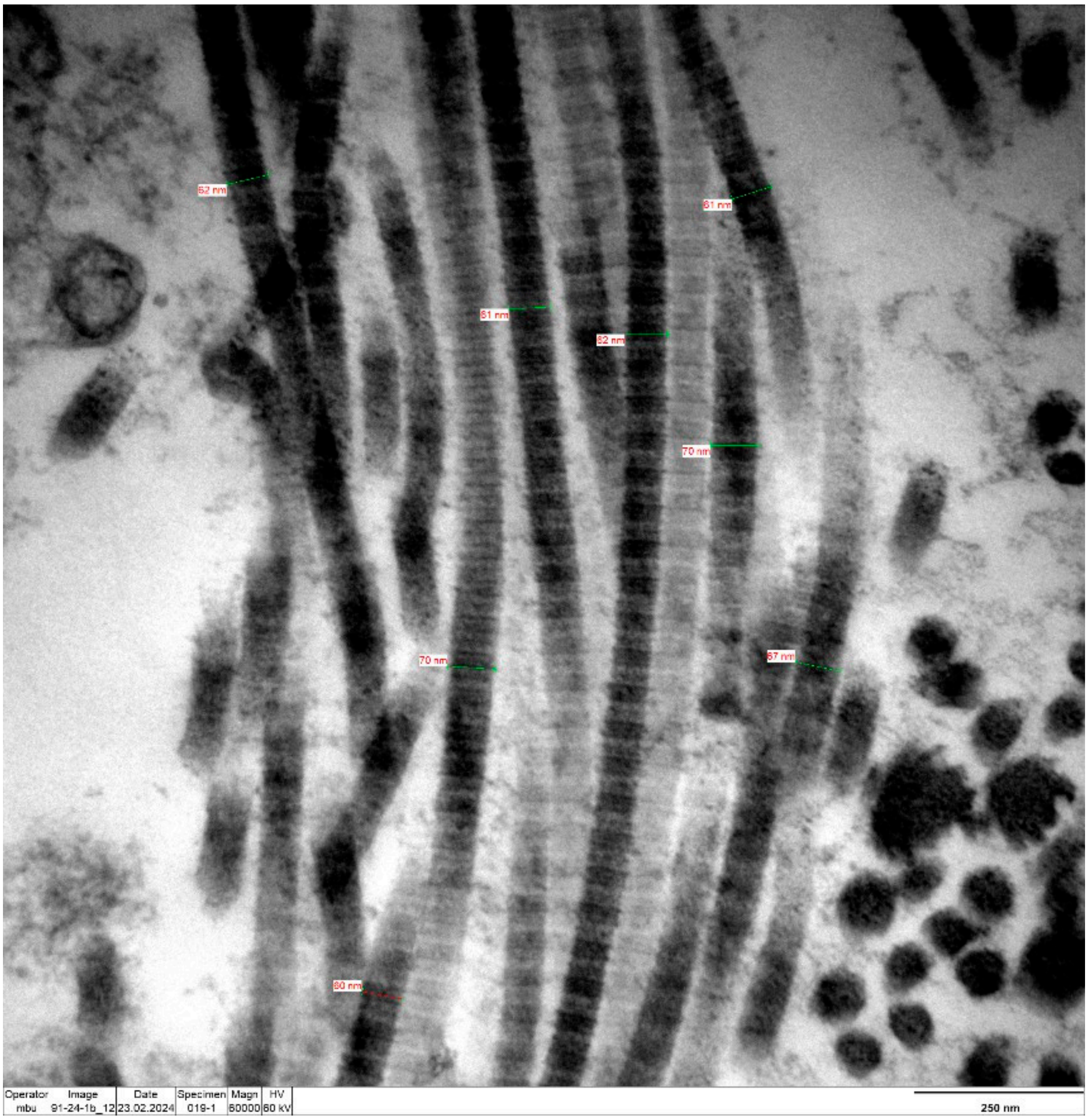

Supplementary Figure S1. Measurement points of the collagen fibers in electron microscopy.
